# The Nervous System in Anæmia

**Published:** 1902-03-29

**Authors:** Louis Vintras

**Affiliations:** Physician to the French Hospital


### Hospital Clinics and Medical Progress.

### THE NERVOUS SYSTEM IN ANÆMIA.

By Louis Vintras, M.D., Physician to the French Hospital.

Anæmia is one of the diseases most commonly met with in everyday practice, and yet one of which we have in no way, at present, a complete understanding. Its ætiology is attributed to that vague class of causes which constitutes the happy hunting ground of medicine, when nothing definite can be found. The disease is looked upon as an abnormal blood condition pure and simple, and iron as its specific treatment. This is anything but satisfactory, and I have for some time been giving more attention to certain concomitant symptoms which are almost invariably found in these cases, than to the condition of the blood *per se*. The result has been that I have gradually come to believe that the nervous system plays a much greater part than is generally recognised, not only in the symptomatology of anæmia, but also in its causation.

If we consider the marked differences presented between what may be called idiopathic anæmia and purely accidental anæmia, we shall better realise the importance assumed by certain symptoms which are generally regarded as secondary. The great majority of cases of anæmia which come under treatment occur in distinctly neurotic subjects. Nervous manifestations accompany the disease from the outset. On inquiry we find evidence of nervous diseases existing in the family. In a case of accidental anæmia, from hæmorrhage, the nervous symptoms only occur late in the disease, and are merely the result of the long continued inadequacy of the blood supply to the nervous centres. The symptoms are typical and belong entirely to the class of nerve prostration; they are also rarely accompanied by hysteria. With idiopathic anæmia we find neuralgia, gastritis, vomiting, painful spasms of the muscles, pains in the peripheral nerves from the beginning; it is indeed one or several of these symptoms, which in many cases first attracts the patient's attention to his or her condition. In young girls, who form by far the largest number of these cases, we often find that menstruation has been painful from its first onset, while the anæmia has appeared only one or two years after this; such cases will present marked hysterical symptoms, a capricious appetite, an unreasonable dislike for the most ordinary articles of food, and a tendency to morbid fancies. In women suffering from anæmia due to a purely accidental cause, amenorrhœa is bound to supervene if the case be a protracted one; but dysmenorrhœa is rarely met with. Green in his "Pathology and Morbid Anatomy" remarks under this disease, that "in many cases the generative organs are also backward in their development." The intimate connection between such a condition of these organs and the existence of nervous diseases is now well established. Finally, as Dr. W. S. Playfair points out in his "Systematic Treatment of Nerve Prostration and Hysteria," profound anæmia always supervenes in cases of nerve prostration. Here the blood condition is undoubtedly produced by the primary nerve trouble.

That there are any distinct nervous lesions even in fatal cases of the disease, a thorough investigation of the question alone can decide, but we must not forget that Banti found degenerative changes in the sympathetic ganglia of the abdomen in pernicious anæmia, and Sasaki similar changes in the nerves of Meistner's and Auerbach's plexus in the intestinal walls.

Coming now to the treatment; it is in cases of idiopathic anæmia that we find that iron so signally fails when employed alone and from the beginning. Under arsenic, strychnine, phosphorus, coca, or any of the many drugs which act directly on the nervous system, especially when seconded by a mild course of electricity, these cases rapidly improve and do well. It is not my wish to discount the value of iron as a means of restoring to the blood the hæmoglobin in which it is deficient; when once the nervous stamina of the patient has been restored, iron and iron alone will complete the cure. But giving iron from the beginning and trusting to it alone is, I contend, attempting to cure a disease by treating a symptom, without first remedying the cause which has produced the condition. What illustrates perhaps better than anything the force of this argument is the recent communication to the Academy of Medicine of Paris, made by Dr. Bouffe, in which he gives the results he has obtained in the treatment of anæmia by intramuscular injections of orchitine. It was quite by chance that Dr. Bouffe made the discovery, while carrying on some investigations as to the value of orchitine in the treatment of psoriasis. He noticed that in several young girls, who were also suffering from anæmia, the anæmia rapidly improved. He immediately pursued his observations in that direction, and is now able to claim for his method most excellent results.

The inference to be deducted from these considerations is obvious. It is in the nervous system that we must look for the primary cause of anæmia in its idiopathic form, and not in any malformation of the heart or arteries as Virchow suggested. The general symptoms point to some interference with the proper innervation of the walls of the blood vessels, and more especially of the capillaries, allowing the corpuscles to linger against the sides and favouring over much their diapedesis; the excess of red corpuscles thus passing into the surrounding tissues perishing and desintegrating. But whatever the ultimate pathology of anæmia may prove to be, it is certain that the successful treatment of the disease depends entirely on our attaching a due importance to its nervous aspects.

